# Overcoming the therapeutic limitations of EZH2 inhibitors in Burkitt’s lymphoma: a comprehensive study on the combined effects of MS1943 and Ibrutinib

**DOI:** 10.3389/fonc.2023.1252658

**Published:** 2023-09-11

**Authors:** Yurim Jeong, Se Been Kim, Chae-Eun Yang, Min Seo Yu, Wan-Su Choi, Youngwoo Jeon, Jung-Yeon Lim

**Affiliations:** ^1^ Department of Biomedical Laboratory Science, Inje University, Gimhae, Republic of Korea; ^2^ Department of Digital Anti-aging Health Care, Inje University, Gimhae, Republic of Korea; ^3^ Department of Hematology, Yeouido St. Mary Hospital, School of Medicine, The Catholic University of Korea, Seoul, Republic of Korea

**Keywords:** Burkitt’s lymphoma, EZH2, Btk, Proteolysis-targeting chimera, miR29b

## Abstract

Enhancer of zeste homolog 2 (EZH2) and Bruton’s tyrosine kinase (BTK) are both key factors involved in the development and progression of hematological malignancies. Clinical studies have demonstrated the potential of various EZH2 inhibitors, which target the methyltransferase activity of EZH2, for the treatment of lymphomas. However, despite their ability to effectively reduce the H3K27me3 levels, these inhibitors have shown limited efficacy in blocking the proliferation of lymphoma cells. To overcome this challenge, we employed a hydrophobic tagging approach utilizing MS1943, a selective EZH2 degrader. In this study, we investigated the inhibitory effects of two drugs, the FDA-approved EZH2 inhibitor Tazemetostat, currently undergoing clinical trials, and the novel drug MS1943, on Burkitt’s lymphoma. Furthermore, we assessed the potential synergistic effect of combining these drugs with the BTK inhibitor Ibrutinib. In this study, we evaluated the effects of combination therapy with MS1943 and Ibrutinib on the proliferation of three Burkitt’s lymphoma cell lines, namely RPMI1788, Ramos, and Daudi cells. Our results demonstrated that the combination of MS1943 and Ibrutinib significantly suppressed cell proliferation to a greater extent compared to the combination of Tazemetostat and Ibrutinib. Additionally, we investigated the underlying mechanisms of action and found that the combination therapy of MS1943 and Ibrutinib led to the upregulation of miR29B-mediated p53-upregulated modulator of apoptosis PUMA, BAX, cleaved PARP, and cleaved caspase-3 in Burkitt’s lymphoma cells. These findings highlight the potential of this innovative therapeutic strategy as an alternative to traditional EZH2 inhibitors, offering promising prospects for improving treatment outcomes in Burkitt’s lymphoma.

## Introduction

Polycomb repressive complex 2 (PRC2) is a transcriptional repressive complex that plays a role in gene silencing through histone methylation (1, 2). Enhancer of zeste homolog 2 (EZH2) is the catalytic subunit of PRC2 and is frequently overexpressed in various cancers, leading to tumorigenesis and poor prognosis (3). Mutations in EZH2, such as Y641 and A677/A687 mutations, enhance its enzymatic activity and promote tumor growth (4). Clinical trials have shown promising results with EZH2-specific inhibitors, including GSK126, EPZ6438, PF-06821497, Tazemetostat and CPI-1205, in treating hematological malignancies and other tumors (5, 6). However, the efficacy of EZH2 inhibitors is limited due to EZH2’s multifunctional roles beyond its catalytic activity in certain cancers, where it can activate genes and contribute to tumor growth through alternative signaling pathways (7, 8). Therefore, targeting these oncogenic functions of EZH2 is crucial for the development of effective therapeutic strategies.

In our study, we employed Proteolysis-targeting chimera (PROTAC) technology, which utilizes a ligand for a protein of interest (POI) and a ligand of E3 ubiquitin ligase connected by a linker to induce degradation of the POI (9, 10). Using this innovative approach, we utilized a hydrophobic tagging strategy with the selective EZH2 degrader, MS1943. Previous studies have demonstrated the remarkable cytotoxic effects of MS1943 specifically in breast cancer cells while preserving the viability of normal cells. Additionally, MS1943 has shown efficacy in *in vivo* models, highlighting its potential as a promising therapeutic agent (11).

Our study specifically focuses on lymphoid malignancies, including B-cell and T-cell lymphomas, where dysregulation of EZH2 is closely linked to unfavorable clinical outcomes (10). Notably, a recently published clinical trial reported an overall response rate (ORR) of 15% for the FDA-approved EZH2 inhibitor Tazemetostat (12). Given these findings, our objective was to evaluate and compare the inhibitory effects of two drugs: the EZH2 inhibitor Tazemetostat (referred to as iEZH2) and the novel PROTAC-based degrader MS1943 (referred to as dEZH2), on different types of lymphoma cells. Through this evaluation, we aimed to assess the efficacy and therapeutic potential of these drugs in the context of lymphoid malignancies.

Furthermore, our study sought to explore the potential synergistic effects of combining these therapies with Ibrutinib, a widely used Bruton’s tyrosine kinase (BTK) inhibitor in Burkitt’s lymphoma treatment (13, 14). The successful application of targeted therapies in cancer treatment has prompted investigations into the potential of BTK inhibitors as a promising approach in specific subsets of lymphomas. BTK, a non-receptor kinase involved in B cell receptor signaling, plays a pivotal role in oncogenic signaling pathways vital for the proliferation and survival of leukemic cells in various B cell malignancies (15). Given the central role of BTK in lymphomagenesis, BTK inhibitors have demonstrated efficacy in B cell malignancies, particularly in subsets of Burkitt’s lymphoma (16). Specifically, Burkitt’s lymphoma cells that exhibit dependency on BCR signaling for growth and survival could benefit from BTK inhibition (17).

In our study, we aimed to explore the synergistic effects of combining the BTK inhibitor Ib with dEZH2 in the context of Burkitt’s lymphoma. By targeting two key factors involved in lymphomagenesis, our approach seeks to leverage the specific vulnerabilities of this subset. Through a comprehensive analysis, we provide insights into the enhanced inhibitory potential of this combination therapy and its underlying mechanisms.

## Methods

### Cell lines and cell culture

In this study, Burkitt’s lymphoma cell lines including RPMI1788, Ramos and Daudi cells were used. These cell lines were obtained from Korean Cell Line Bank (Seoul, Republic of Korea). These cell lines were grown in RPMI 1640 medium (Gibco, Carlsbad, CA, USA) supplemented with 10% heat-inactivated fetal bovine serum (FBS; Gibco), 2mM L-glutamine (Gibco), 1% antibiotics (10 U/mL penicillin and 10 g/mL streptomycin; Gibco). All cell lines were incubated at 37°C and 5% CO_2_.

### Drug preparations

Tazemetostat (iEZH2, EZH2 inhibitor), MS1943 (dEZH2, EZH2 degrader), and Ibrutinib (Ib, BTK inhibitor) were purchased from MedChemExpress (NJ, USA). These drugs were dissolved in dimethyl sulfoxide (DMSO) as recommended by the manufacturer and stored at -80°C. All these drugs were diluted with the RPMI 1640 medium with 10% heat-inactivated FBS, 2mM L-glutamine and 1% antibiotics before used for the treatment of cell lines.

### Cell proliferation assay

Cell growth was assessed using the CCK-8 (Dojindo, Rockville, MD, USA) assay according to the manufacturer’s protocol. RPMI1788, Ramos, and Daudi cell lines were seeded at an initial cell density of 2 × 10^4^ cells/100μL culture medium in 96-well plates. Different doses of Tazemetostat, MS1943, and Ibrutinib drugs were administered to the cells, including concentrations of 2.5μM, 5μM, and 10μM for each drug. Additionally, cells were treated with combinations of these drugs or left untreated as controls. Cultures were maintained at 37°C in a 5% CO_2_ atmosphere. CCK-8 solution was added 10μL to each well after 24 hours, 48 hours and 72 hours. The plates were incubated for 1-4 hours in a CO_2_ incubator, and the optical density was measured at 450nm using a microplate reader. Cell viability is calculated by OD of test group/OD of control × 100%. The combination index was evaluated by CalcusynTM software. All the experiments were performed at triplicate.

### Western blotting

One million RPMI1788, Ramos or Daudi cells were lysed in 2× laemmli sample buffer (Bio-Rad) with β-mercaptoethanol and boiled at 95°C for 10 min. After removal of the insoluble fraction by centrifugation at 10,000 ×g for 10 min, protein samples were separated by SDS gel electrophoresis and transferred to a polyvinylidene difluoride membrane. Membranes were stained with cleaved PARP, PARP, cleaved caspase-3, PUMA, XIAP and p53 antibodies (Cell Signaling Technology, MA, USA) at a dilution of 1: 1,000 or GAPDH and β-actin antibody (Cell Signaling Technology) at a dilution of 1: 5,000 at 4°C overnight. After overnight incubation in 4°C, HRP-conjugated secondary antibody was added. After washing with Tris-buffered saline and Tween 20, the hybridized bands were detected using an enhanced chemiluminescence (ECL) detection kit (Amersham Pharmacia Biotech, Buckinghamshire, UK).

### Mir29B silencing with siRNAs

A pool of nontargeting siRNAs and a pool of mir29B-targeting siRNAs were purchased from Thermo Fisher Scientific. Cells were plated in 24-well plates at 5 × 10^5^ cells per well for 72 hrs before transfection. Lipid–siRNA complexes were prepared by incubating 50 nM of siRNA with lipofectamine RNAiMAX (Life Technologies) in the volume recommended by the manufacturer. Lipid–siRNA complexes were added to the wells in a final volume of 1 mL of serum-free RPMI 1640. After incubation for 2 hrs, cells were reincubated with or without drugs in RPMI 1640 containing 10% heat-inactivated FBS.

### RNA extraction and cDNA synthesis

The 5 × 10^5^ cells treated with or without drugs same as described. Total RNA was extracted using TRIzol reagent (Invitrogen) and the concentration of RNA was measured using the NanoDrop. These were reverse transcribed using High Capacity RNA-to-cDNA Kit (Appliedbiosystems, MA, USA) following to the manufacturer’s protocol. The cDNA was synthesized using Thermocycler (Bio-Rad, CA, USA) with cycling conditions used include primer annealing at 25°C for 10 minutes, DNA polymerization at 37°C for 120 minutes and finally reverse transcriptase deactivation at 85°C for 5 minutes. The synthesized cDNA was stored at -20°C before further use.

### Real-time reverse transcription PCR

RT-PCR was performed using the TB Green® Fast qPCR Mix (TaKaRa Bio, Japan) using the manufacturer’s protocols: Hold 1 cycle for 30 seconds at 95°C, 2 step PCR 40 cycles for 5 seconds at 95°C and 30 seconds at 60°C. Add the Dissociation steps consisting of 15 seconds at 95°C, 30 seconds at 60°C and 15 seconds at 95°C for 1 cycle. For detection the mechanisms of the treated drugs in Burkitt’s lymphoma, the follbowing gene-specific primers were used: Chop (forward: *5’-GCACCTCCCAGAGCCCTCACTCTCC-3’*; reverse: *5’-GTCTACTCCAAGCCTTCCCCCTGCG-3’*), Bip (forward: *5’-CGAGGAGGAGGACAAGAAGG-3’*; reverse: *5’-CACCTTGAACGGCAAGAACT-3’*), cyclin D1 (sense: *ATGCCAACCTCCTCAACGAC*; antisense: *GGCTCTTTTTCACGGGCTCC*), Xbp1 (forward: *5’- TGCTGAGTCCGCAGCAGGTG -3’*; reverse: *5’- GCTGGCAGGCTCTGGGGAAG -3’*), β-actin (forward: *5’- TCACCCACACTGTGCCCATCTACGA -3’*; reverse: *5’- AGCGGAACCGCTCATTGCCAATG -3’*), and GAPDH (forward: *5’-CCACTCCTCCACCTTTGACG-3’*; reverse: *5’-CCACCACCCTGTTGCTGTAG -3’*). Multiplex quantitative PCR was performed using the following TaqMan gene expression assays: mir28B (Hs04231440) and GAPDH (Hs99999905), all from Thermo Fisher Scientific. For quantification, relative mRNA expression of specific genes was calculated using the 2-^ΔΔCt^ method, after normalization to GAPDH or β-actin expression.

### Cell cycle analysis

The 5 × 10^5^ cells were treated with the drugs described. After incubating for 72 hours in CO_2_ incubator, the cells were harvested and centrifuged 2,000rpm 5 minutes with DPBS (Gibco). These cells were performed twice for wash and discarded the supernatant. For cell cycle analysis, the Annexin V-PI Apoptosis Kit (BioVision, London, UK) were used following the recommended methods. The cell cycle was detected using the BD LSRFortessa (MA, USA).

### Statistical analysis

Each experiment was repeated at least three times to ensure the reproducibility of the results. Statistical significance was determined using Student’s two-tailed t-test and one-way analysis of variance (ANOVA) with Bonferroni correction for multiple comparisons. Differences between expression levels at a given time point were evaluated by χ2 contingency analysis. In all analyses, P values less than 0.05 were considered to indicate statistical significance.

## Results

### MS1943 (dEZH2) exhibits stronger anti-proliferative effects and induces prolonged activation of the UPR pathway compared to Tazemetostat (iEZH2) in Burkitt’s lymphoma

**Figure 1:**
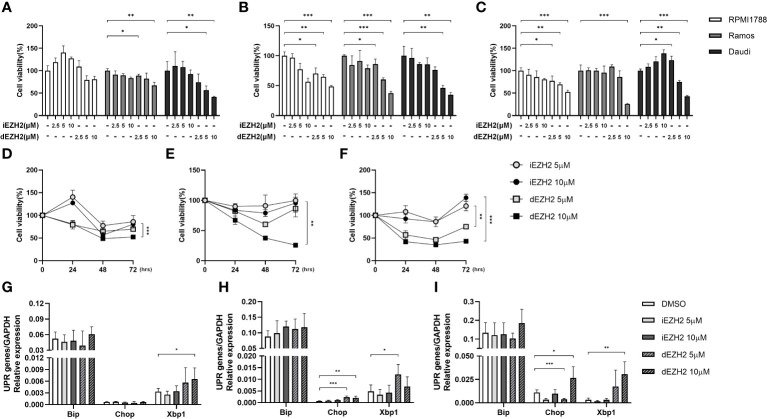
Time- and concentration-dependent effects of iEZH2 and dEZH2 in Burkitt’s lymphoma. **(A–C)** RPMI1788, Ramos, Daudi cells were treated with 2.5 μM, 5 μM and 10 μM of iEZH2 or dEZH2, along with DMSO as a control, for 24 **(A)**, 48 **(B)** and 72 **(C)** hours. Cell viability was assessed using a CCK-8 assay and compared to the control at each concentration. **(D–F)** The time-dependent effect on cell viability was examined in RPMI1788 **(D)**, Ramos **(E)** and Daudi **(F)** cell lines treated with 5 μM or 10 μM of iEZH2 or dEZH2. **(G–I)** The relative mRNA expression of genes associated with the UPR was determined by qPCR. RPMI1788 **(G)**, Ramos **(H)** and Daudi **(I)** cell lines treated with 5 μM and 10 μM of iEZH2 or dEZH2 for 48 hours. The expression levels at each concentration were compared with control, and each target gene was normalized to GAPDH. Statistical testing was conducted with two-tailed, unpaired t-tests, *, *p <*0.05; **, *p <*0.01; ***, *p <*0.001. Error bars represent the ± SD.

To assess the inhibitory effects of iEZH2 and dEZH2 on various lymphoma cell lines, we treated the cells with different concentrations of each drug at various time points and evaluated their impact on cell viability using the CCK-8 assay. Our results demonstrated that dEZH2 exhibited significantly greater inhibitory effects than iEZH2 in all lymphoma cell lines tested (data not shown), particularly in Burkitt’s lymphoma including RPMI1788, Ramos, and Daudi cells at 24hrs (**Figure 1A**), 48hrs (**Figure 1B**), and 72 hours (**Figure 1C**) of drug treatment, in a dose-dependent manner (**Figures 1D–F**).

Furthermore, dEZH2 is known to induce cell death through the unfolded protein response (UPR) pathway-mediated endoplasmic reticulum (ER) stress (11). To investigate this mechanism in Burkitt’s lymphoma, we examined the expression of downstream effectors of the UPR pathway, including Xbp1, Chop, and Bip, using qPCR. Remarkably, treatment with dEZH2 resulted in increased expression of these factors in Burkitt’s lymphoma (**Figures 1G–I**), with a significant upregulation observed in Daudi cells (**Figure 1I**), which exhibited the most pronounced inhibitory effects (**Figures 1A–C**). Also, these findings suggest a correlation between the observed anti-proliferative effects and the differential expression of Xbp1, Chop, and Bip.

Collectively, our results provide compelling evidence that dEZH2 exhibits a pronounced inhibitory effect on Burkitt’s lymphoma compared to iEZH2, highlighting its potential as a promising therapeutic agent in the treatment of Burkitt’s lymphoma.

### Combination therapy with Ib (BTK inhibitor, Ibrutinib) and dEZH2 synergistically inhibits the proliferation of Burkitt’s lymphoma *in vitro*

Based on our assumption that the BTK inhibitor Ib, which can be used in some subsets of Burkitt’s lymphoma, could have a synergistic effect when combined with dEZH2, we tested the combination therapy of Ib with iEZH2 or Ib with dEZH2. Initially, we treated various Burkitt’s lymphoma cell lines with 5 μM of Ib, iEZH2, dEZH2, either alone or in combination, and assessed cell viability over time using the CCK-8 assay. Regarding the selection of drug concentrations, we have determined the IC50 values for MS1943 in different cell lines. The IC50 values for RPMI1788 cell, Ramos, and Daudi cell line were found to be 9.017 μM, 8.425 μM, and 6.076 μM, respectively (**Supplementary Data 1**).

**Figure 2:**
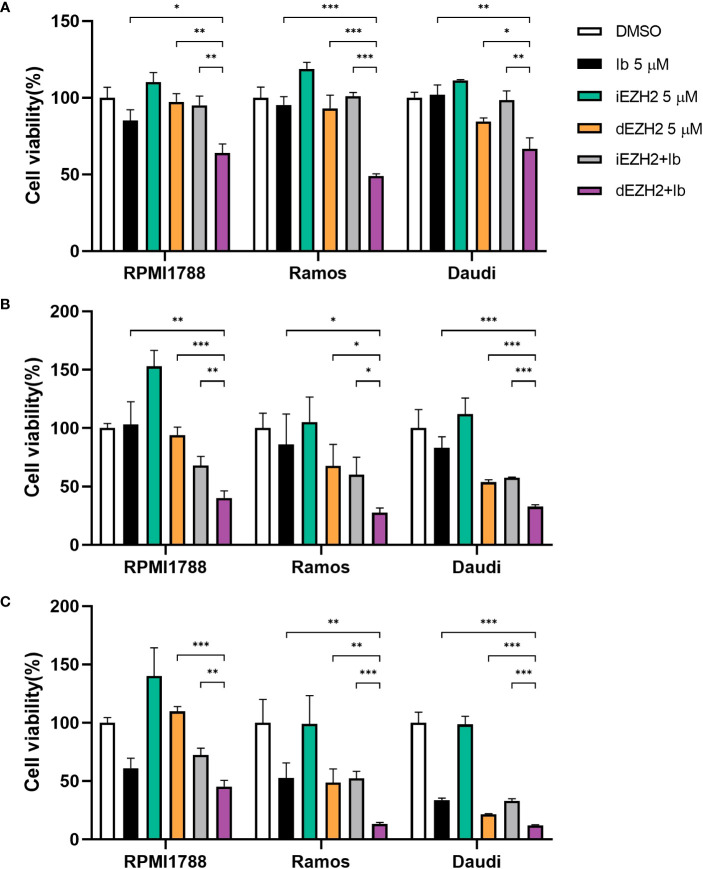
Combination therapy of Ib with iEZH2 or dEZH2 in Burkitt’s lymphoma. **(A–C)** Cell viability was analyzed by CCK-8 assay in RPMI1788, Ramos and Daudi cell lines. All cell lines were plated at 2×10^4^/well and exposed to 5 μM of each drug or combination of Ib and iEZH2 or Ib and dEZH2 for 24 **(A)**, 48 **(B)** and 72 **(C)** hours. At the end of incubation, 10 μL/well of the CCK-8 reagent was added to each well. Detection was performed at 450nm using spectrophotometer after 4 hours. Statistical testing was conducted with two-tailed, unpaired t-tests, *, *p <*0.05; **, *p <*0.01; ***, *p <*0.001. Error bars represent the ± SD.

Notably, combination of Ib and dEZH2 therapy demonstrated significant inhibitory effects across all Burkitt’s lymphoma cell lines at 24, 48, and 72 hours (**Figures 2A–C**), with the most prominent cytotoxicity observed after 72 hours of culture, relative to the single-treated groups (**Figure 2C**). To calculate the combination index (CI), CalcuSyn software was used. The CI values are characterized by synergistic (CI < 0.9), additive (CI = 1), and antagonistic (CI > 1). As shown, the combination of Ib and dEZH2 revealed a CI value of < 1 signifying a synergistic mechanism of action (**Supplementary Data 2**).

In conclusion, iEZH2 treatment was ineffective in Burkitt’s lymphoma, despite the importance of EZH2 in the disease (10). However, the combination of dEZH2 with the established lymphoma therapeutic agent, Ib, showed significant therapeutic effects compare to iEZH2 with Ib (**Figure 2**). This highlights the potential of dEZH2 as an effective treatment option for Burkitt’s lymphoma, overcoming the limitations of conventional iEZH2 treatment.

### Combination therapy with Ib and dEZH2 induces G2/M-phase arrest in Burkitt’s lymphoma *in vitro*

**Figure 3:**
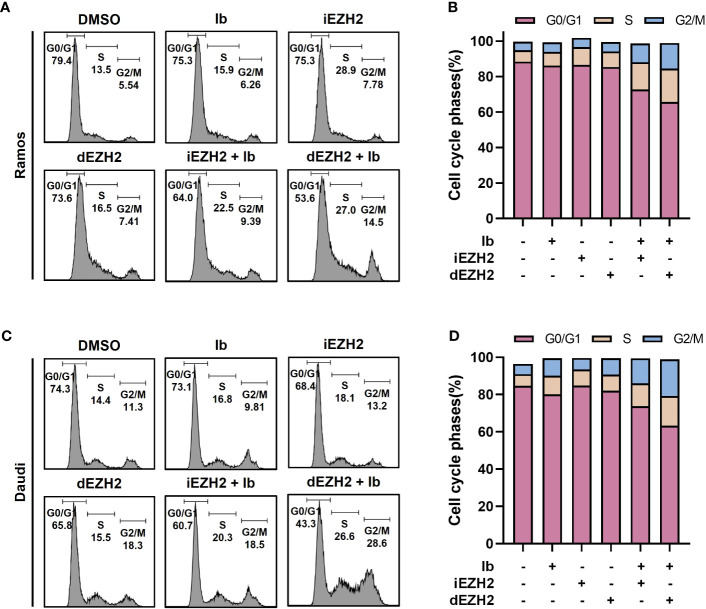
Combination treatment with Ib and dEZH2 resulted in an increased G2/M phase arrest, as assessed by flow cytometry analysis of cell cycle phases. The ratio of cell cycle phases was measured by flow cytometry. **(A)** In the Ramos cell line, the major peaks corresponded to the G0/G1 phase, S phase, and G2/M phase from left to right. **(B)** Ramos cells were treated with 5 μM of Ib, iEZH2, and dEZH2 for 72 hours. **(C)** The cell cycle analysis in the Daudi cell line followed the same order as the Ramos cell line. Daudi cells were treated under equivalent conditions as Ramos cells. **(D)** The cell cycle phases were expressed as a percentage ratio, excluding a few cells outside the range. Statistical testing was conducted with two-tailed, unpaired t-tests. Error bars represent the ± SD.

To investigate the correlation between cell proliferation inhibition and cell cycle arrest, flow cytometric analysis was performed to assess cell cycle distribution. Treatment with a combination of Ib and dEZH2 resulted in an increase in the number of cells in the G2/M phase after 72 hours of treatment, accompanied by a decrease in the number of cells in the G0/G1 phase in Ramos (**Figures 3A, B**) and Daudi cells (**Figure 3D**).

### Cell apoptosis induced by Ib combined with dEZH2 in Burkitt’s lymphoma

To investigate the underlying mechanisms behind the synergistic inhibitory effects of Ib and dEZH2 in Burkitt’s lymphoma, we conducted flow cytometric analysis. Among the lymphoma cell lines demonstrating significant inhibitory effects, Ramos and Daudi cells were selected for further investigation. These cells were treated with Ib, iEZH2, and dEZH2 either alone or in combination at a concentration of 5 μM for 72 hours.

**Figure 4:**
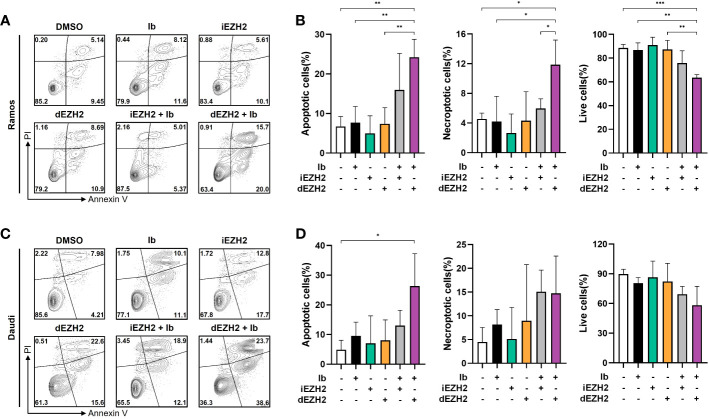
A synergy effect of apoptosis was observed in co-treatment of Burkitt’s lymphoma. **(A)** Annexin V/PI staining was used to determine the apoptotic effect in Ramos cells. Cells were treated with individual or combinations of 5 μM of Ib, iEZH2, and dEZH2 for 72 hours. **(B)** The percentages of apoptotic cells (Annexin V-positive/PI-negative), live cells (Annexin V-negative/PI-negative) and necroptotic cells (Annexin V-positive/PI-positive) were compared with control (DMSO). **(C)** Daudi cells were treated with 5 μM of Ib, iEZH2 and dEZH2, either individually or in combination for 72 hours. **(D)** The percentage of apoptotic cells, live cells and necroptotic cells in Dauid cells were compared to the control. Statistical testing was conducted with two-tailed, unpaired t-tests, *, *p <*0.05; **, *p <*0.01; ***, *p <*0.001. Error bars represent the ± SD.

Strikingly, the combination treatment of Ib and dEZH2 led to a notably higher proportion of apoptotic and necroptotic cells compared to either individual treatment or the combination of Ib and iEZH2. Furthermore, the population of live cells significantly decreased in the group receiving the combination therapy of Ib and dEZH2 (**Figures 4A–D**). These findings strongly suggest that the combined administration of Ib and dEZH2 enhances apoptotic and necroptotic cell death, resulting in a significant reduction in viable cells.

### Combination treatment of Ib and dEZH2 causes apoptosis through p53-dependent pathway in Burkitt’s lymphoma

**Figure 5:**
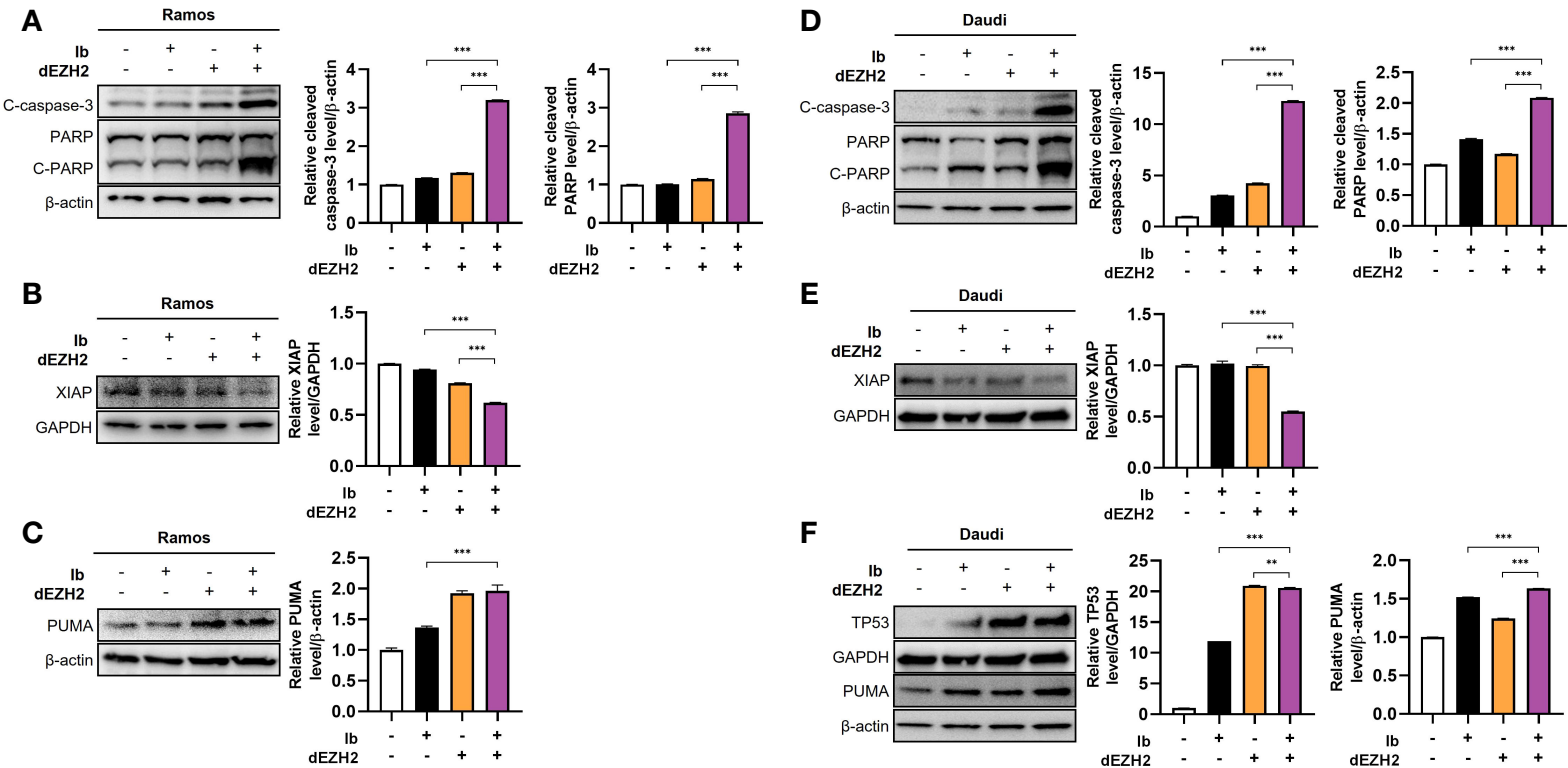
Enhanced levels of apoptosis-associated proteins were observed in Burkitt’s lymphoma treated with combination therapy. Western blot analysis was performed to examine the protein expression in Ramos and Daudi cells treated with 5 μM of Ib, dEZH2, or the combination. **(A)** In the Ramos cell line, the protein levels of cleaved caspase-3 (C-caspase-3), PARP, and cleaved PARP (C-PARP) were compared to β-actin. The total form of PARP (116 kDa) and C-PARP (89 kDa) were detected using a primary antibody. **(B)** The protein level of XIAP was normalized to GAPDH. **(C)** The protein level of PUMA, which is associated with p53, was measured relative to β-actin. **(D)** Similar to the Ramos cell line, the Daudi cell line showed increased protein levels of C-caspase-3, PARP, and C-PARP relative to β-actin. **(E, F)** The protein level of XIAP was compared to GAPDH, and the relative protein levels of p53 and PUMA were determined relative to GAPDH and β-actin, respectively. Statistical testing was conducted with two-tailed, unpaired t-tests, **, *p <*0.01; ***, *p <*0.001. Error bars represent the ± SD.

After confirming the induction of apoptosis upon simultaneous inhibition of Ib and dEZH2, we investigated the association with specific apoptosis-related pathways through western blot analysis. PARP is a crucial protein involved in DNA repair, and upon activation by caspase-3 cleavage, it transforms into its cleaved form, leading to DNA damage and apoptosis (18, 19). Comparing to the control group, we observed an increase in the protein levels of cleaved PARP and its active form, cleaved caspase-3 in Ramos (**Figure 5A**) and Daudi cells (**Figure 5D**). Furthermore, X-linked inhibitor of apoptosis (XIAP), a protein that inhibits caspase-3 activity, exhibited decreased expression in the combination treatment group (**Figures 5B, E**), further supporting our previous findings (20, 21).

Next, we explored the association with the well-known pro-apoptotic gene, TP53. TP53 induces cell cycle arrest by damaging DNA, preventing cell proliferation, and activates p53 upregulated modulator of apoptosis (PUMA), which promotes cell death in cancer cells (22, 23). In both cell lines, the combination treatment resulted in an increase in the expression of TP53 and PUMA (**Figures 5C, F**). Collectively, these findings demonstrate that this drug combination enhances p53-dependent apoptosis in Burkitt’s lymphoma.

In conclusion, our study provides evidence that this drug combination increases p53-dependent apoptosis in Burkitt’s lymphoma, as evidenced by the upregulation of cleaved PARP, cleaved caspase-3, TP53, and PUMA.

### miR29b is essential factor of anti-tumor effect in combination therapy with Ib and dEZH2

p53 plays an essential role in regulating microRNAs (miRNAs), which are critical for gene expression control (24). Activation of TP53 promotes the expression of miRNAs, contributing to either tumor suppression or promotion (25). Specifically, miR29b has been implicated in activating p53 and acting as a downstream pathway of miR29b (25). Building upon these findings, we conducted qPCR to investigate whether our results were mediated by miR29b pathway.

**Figure 6:**
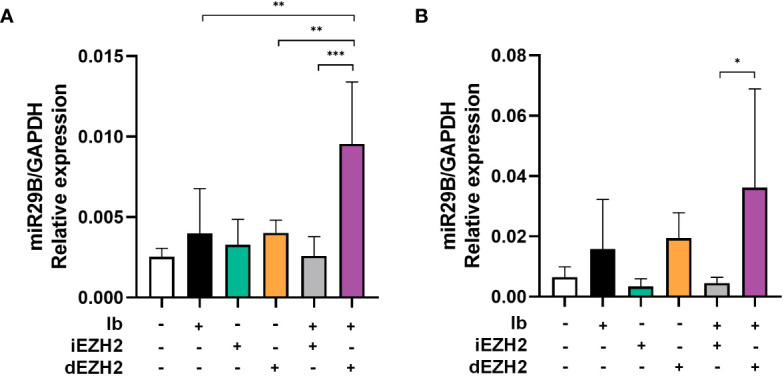
Upregulation of miR29B was observed in Burkitt’s lymphoma treated with both Ib and dEZH2. **(A, B)** Upregulation of miR29B was observed in Burkitt’s lymphoma treated with both Ib and dEZH2. Ramos **(A)** and Daudi **(B)** cell lines were incubated for 72hours after treatment with Ib, iEZH2 and dEZH2. The levels of miR29B were normalized to GAPDH. Statistical testing was conducted with two-tailed, unpaired t-tests, *, *p <*0.05; **, *p <*0.01; ***, *p <*0.001. Error bars represent the ± SD.

Remarkably, when treated with a combination of Ib and dEZH2, we observed a significant increase in miR29b expression in Ramos (**Figure 6A**) and Daudi cells (**Figure 6B**) compared to the single groups.

**Figure 7:**
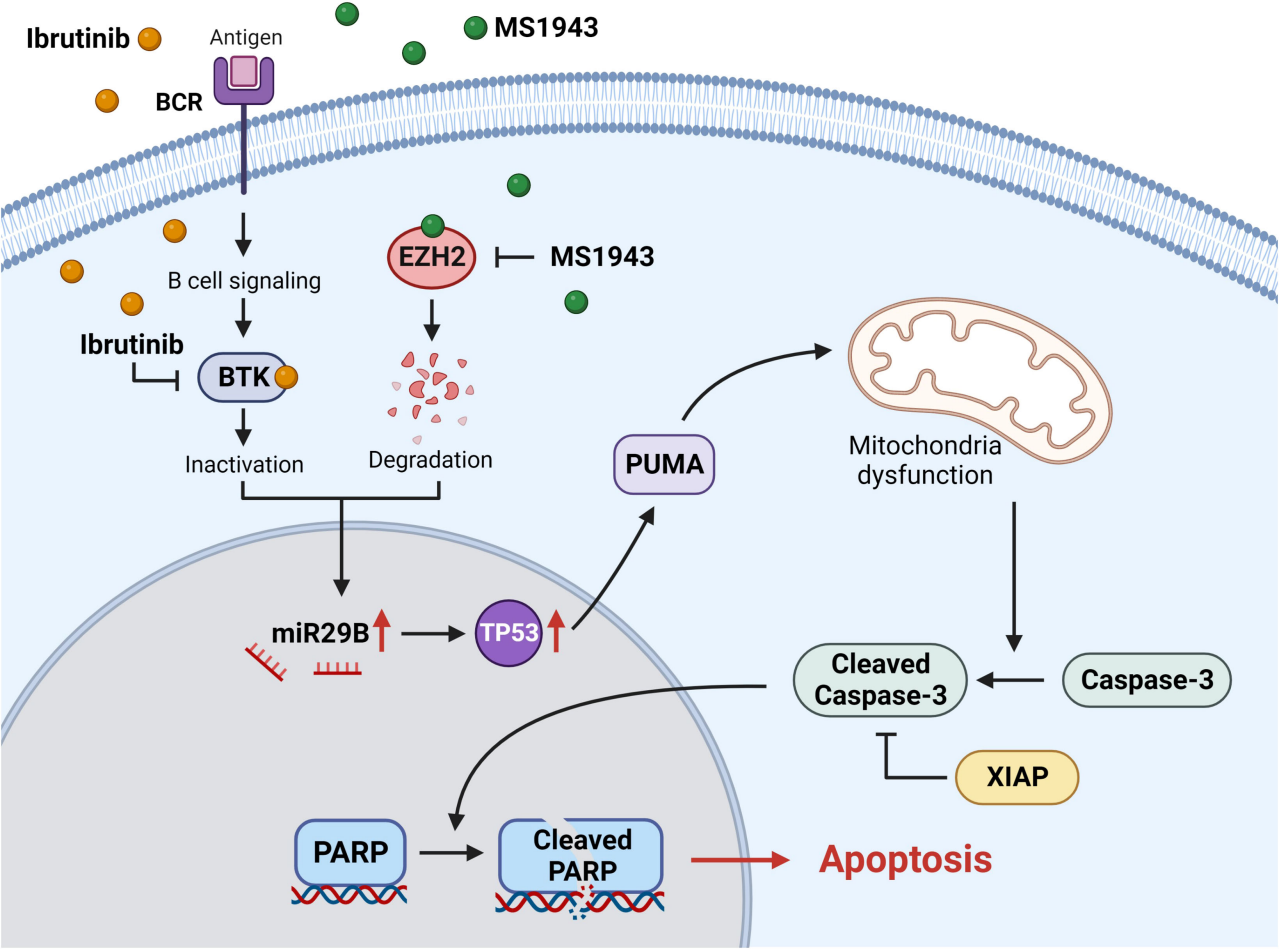
The signaling pathway of miR29B-mediated apoptosis involves the degradation of EZH2 and the inhibition of BTK. Ibrutinib effectively inhibits and inactivates BTK, a critical component of the B-cell receptor signaling (BCR) pathway. On the other hand, MS1943 acts as a potent EZH2 degrader. Simultaneous induction of these reactions leads to the upregulation of miR29B, which in turn activates the downstream pathway involving TP53. The increased levels of TP53 promote the expression of PUMA, a pro-apoptotic protein, ultimately leading to cell death. The activation of PUMA induces mitochondrial dysfunction through a series of intricate processes. Subsequently, caspase-3 is activated and cleaved, triggering the cleavage of PARP within the cell nucleus. This cascade of events ultimately culminates in apoptosis. It is worth noting that XIAP, an anti-apoptotic protein, inhibits the activity of cleaved caspase-3.

Overall, our findings provide evidence that the simultaneous treatment of Ib and dEZH2 in Burkitt’s lymphoma induces apoptosis through the miR29b-mediated TP53 upregulated pathway, thereby exhibiting potent anti-tumor effects (**Figure 7**).

## Discussion

Lymphoma is a type of cancer that originates in the lymphatic system, characterized by the abnormal growth of white blood cells, and ranks as the sixth most prevalent cancer worldwide, excluding non-melanoma skin cancer (26). Lymphoma is associated with a poor prognosis due to its heterogeneous nature and variable treatment responses. Given the aggressive behavior and risk of relapse, there is a critical need for novel therapeutic strategies to achieve lasting remissions and improve patient outcomes (27). Understanding the underlying molecular mechanisms and developing targeted therapies are essential in addressing the challenges posed by lymphoma.

EZH2 plays a significant role in lymphoma as the catalytic subunit of PRC2, regulating gene expression through histone methylation (28). Its aberrant expression and function are associated with lymphomagenesis and have led to the development of therapeutic inhibitors. Understanding the diverse mechanisms by which EZH2 contributes to lymphoma pathogenesis, including epigenetic regulation and interactions with the tumor microenvironment, provides valuable insights for targeting EZH2 in lymphoma treatment (28, 29).

Despite being recognized as an important target in lymphoma, the FDA-approved small molecule inhibitor tazemetostat targeting EZH2 has not yielded the expected outcomes. Its efficacy has been found to be suboptimal not only in lymphoma but also in other cancer types.

MS1943, a PROTAC-based drug, demonstrated remarkable efficacy in suppressing T-cell lymphoma, B-cell lymphoma, Hodgkin’s lymphoma, and non-Hodgkin’s lymphoma cell lines compared to the conventional tazemetostat drug (data not shown). Importantly, the extent of inhibition was found to be correlated with the expression levels of UPR pathway-associated genes, including Bip, Chop, and Xbp1, showing higher expression in MS1943-treated samples compared to the control group. In our study, we observed that Daudi cells exhibited the most pronounced inhibitory effects when treated with MS1943 (**Figures 1A–F**), and the expression of UPR-associated genes, namely Bip, Chop, and Xbp1, was significantly upregulated (**Figure 1G**). These findings are consistent with previous studies in breast cancer, where triple-negative breast cancer (TNBC) cell line showing responsiveness to MS1943 treatment were also characterized by increased expression of UPR-related genes (11). This suggests a potential association between the observed inhibitory effects of MS1943 and the expression of UPR pathway genes, highlighting the significance of UPR signaling in the response to MS1943 treatment.

In addition, our study focused on investigating the potential therapeutic efficacy of simultaneous targets of EZH2 and BTK in Burkitt’s lymphoma and exploring the underlying apoptotic pathways associated with this drug combination. Burkitt’s lymphoma is a heterogeneous group of malignancies characterized by dysregulation of various signaling pathways (30), making it challenging to develop effective treatments. Targeting multiple pathways simultaneously may offer a promising approach to overcome these challenges.

Our findings demonstrated that the combination treatment of MS1943 with Ibrutinib resulted in a significant induction of apoptosis in Burkitt’s lymphoma cell lines, specifically Ramos and Daudi cells (**Figure 4**). Apoptosis, or programmed cell death, is a crucial process for maintaining tissue homeostasis and preventing the proliferation of damaged or cancerous cells (31). Therefore, promoting apoptosis is a desirable therapeutic strategy.

Western blot analysis revealed increased protein levels of cleaved PARP and cleaved caspase-3, both of which are markers of apoptosis, in the combination treatment group compared to the control group (**Figures 5A, D**). This observation suggests that the simultaneous inhibition of EZH2 and BTK leads to the activation of caspase-dependent apoptotic pathways. The cleavage of PARP and caspase-3 is an irreversible event in the execution phase of apoptosis, further confirming the apoptotic response induced by the drug combination.

Furthermore, the downregulation of XIAP, an inhibitor of caspase-3 activity, in the combination treatment group further supports the involvement of apoptotic pathways in the observed therapeutic effects (**Figures 5B, E**). XIAP is a member of the inhibitor of apoptosis (IAP) family, which plays a critical role in blocking apoptosis by inhibiting caspases (32, 33). The downregulation of XIAP in response to the drug combination suggests a release of caspase inhibition, promoting apoptosis.

Moreover, our study investigated the association between the drug combination and the tumor suppressor protein TP53, which plays a crucial role in regulating apoptosis (34, 35). We observed an upregulation of TP53 and its downstream target PUMA (TP53 upregulated modulator of apoptosis) in response to the combination treatment in Daudi cells (**Figures 5C, F**). These results suggest that the enhanced apoptosis induced by the drug combination is mediated through TP53-dependent mechanisms.

The activation of TP53 and the subsequent upregulation of PUMA lead to the initiation of cell cycle arrest and promotion of cell death in B-cell lymphoma cells (36). The induction of G2/M-phase cell cycle arrest observed in our study further supports the involvement of cell cycle regulatory pathways in the apoptotic response to the drug combination (**Figure 3**).

Furthermore, our study explored the potential role of miRNA regulation in the observed apoptotic response. TP53 has been implicated in the regulation of miRNA expression (24), which can either promote or suppress tumor growth depending on the context. Specifically, miR29b has been reported to activate TP53 and act as a downstream mediator of p53’s tumor-suppressive functions (24, 37). miR29b plays a pivotal role in cancer by regulating various processes. It targets Myeloid cell leukemia-1 (Mcl-1) to modulate apoptosis and enhance sensitivity to cytotoxic treatments (38). Moreover, miR-29b impacts cell proliferation, apoptosis, and differentiation by targeting AKT2, cyclin D2 (CCND2), and genes associated with osteoclastic differentiation (38). These findings highlight miR-29b’s diverse functions in cancer and related pathways.

Based on this knowledge, we investigated the expression of miR29b in response to the drug combination. Our qPCR analysis revealed a significant increase in miR29b expression in Ramos and Daudi cells treated with Ibrutinib and MS1943, supporting its involvement in the observed apoptotic response (**Figure 6**).

In conclusion, our study provides compelling evidence for the therapeutic potential of simultaneous EZH2 and BTK targets in Burkitt’s lymphoma. The observed induction of apoptosis and the involvement of TP53-dependent pathways highlight the importance of targeting multiple signaling pathways for effective treatment strategies. These findings contribute to the understanding of the molecular mechanisms underlying Burkitt’s lymphoma pathogenesis and offer insights into the development of novel combination therapies for improved clinical outcomes in Burkitt’s lymphoma patients. Further studies are warranted to elucidate the detailed mechanisms and evaluate the therapeutic potential of this drug combination in preclinical and clinical settings. The development of personalized treatment strategies based on targeting specific pathways holds promise for the future management of Burkitt’s lymphoma.

## Data availability statement

The raw data supporting the conclusions of this article will be made available by the authors, without undue reservation.

## Ethics statement

Ethical approval was not required for the studies on humans in accordance with the local legislation and institutional requirements because only commercially available established cell lines were used.

## Author contributions

J-YL and YoJ conceived the study. YuJ, C-EY, SK, MY, WC, YoJ, and J-YL conducted experiments and analyzed data. YuJ, SK, and J-YL wrote the manuscript with input from all authors.
