# Supplementary figures and images for: Overcoming the therapeutic limitations of EZH2 inhibitors in Burkitt’s lymphoma: a comprehensive study on the combined effects of MS1943 and Ibrutinib
Source: Front Oncol. 2023 Sep 11;13:1252658. doi: 10.3389/fonc.2023.1252658 (PMC10518396; doi:10.3389/fonc.2023.1252658)

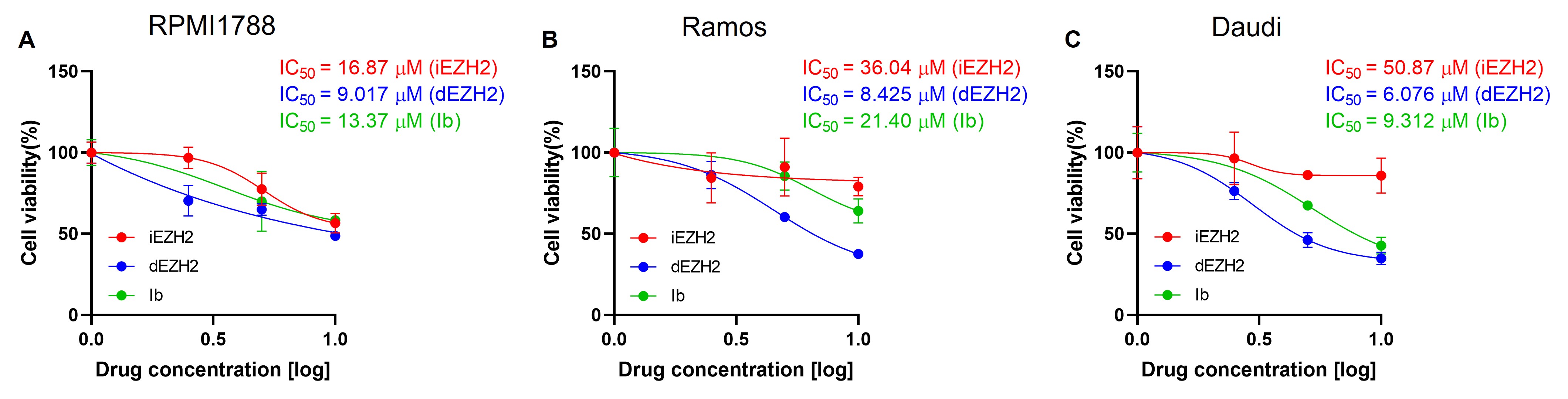

Supplement: Supplementary file 1 [file Image_1.jpeg]

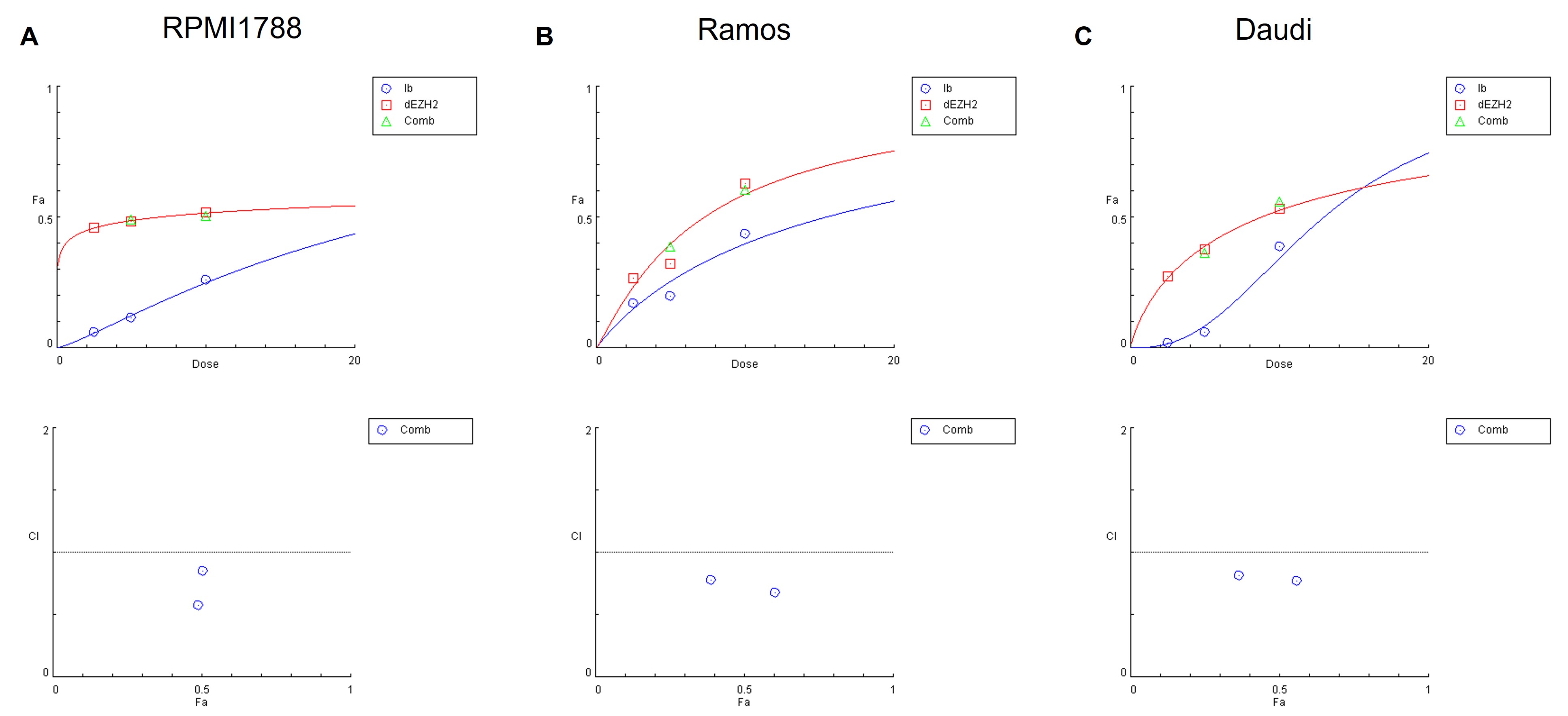

Supplement: Supplementary file 2 [file Image_2.jpeg]
